# Association of High Immunohistochemical Expression of Minichromosome Maintenance 3 with Human Oral Squamous Cell Carcinoma—A Preliminary Study

**DOI:** 10.3390/diagnostics13010061

**Published:** 2022-12-26

**Authors:** Rabia Zahir, Zafar Ali Khan, Benish Aleem, Shahzad Ahmad, Asif Ali, Rakhi Issrani, Mohammed Katib Alruwaili, Shazia Iqbal, Shmoukh Fahad Alghumaiz, Sarah Hatab Alanazi, Muhammad Farooq Umer, Ihsan Ullah, Sumera, Kiran Kumar Ganji

**Affiliations:** 1 Institute of Basic Medical Sciences, Khyber Medical University, Peshawar 25100, Pakistan; 2 Department of Oral & Maxillofacial Surgery and Diagnostic Sciences, College of Dentistry, Jouf University, Sakaka 72388, Saudi Arabia; 3 Institute of Pathology & Diagnostic Medicine, Khyber Medical University, Peshawar 25100, Pakistan; 4 Department of Oral Medicine & Diagnostic Sciences, Vision College, Riyadh 0096611, Saudi Arabia; 5 Institute of Cancer Sciences, University of Glasgow, Glasgow G12 8QQ, UK; 6 Department of Preventive Dentistry, College of Dentistry, Jouf University, Sakaka 72388, Saudi Arabia; 7 Department of Periodontology and Endodontology, Faculty of Dental Medicine, Hokkaido University, N13W7, Kita-ku, Sapporo, Hokkaido 060-8586, Japan; 8 Department of Obstetrics & Gynecology, Vision College, Riyadh 0096611, Saudi Arabia; 9 Sayyaf Dental Complex, Sakaka 72311, Saudi Arabia; 10 The Golden Al-Miswak Dental Complex, Al Qrayyat 75911, Saudi Arabia; 11 Alshifa School of Public Health, Rawalpindi 46200, Pakistan; 12 Department of Periodontology and Implantology, Sharad Pawar Dental College & Hospital, Datta Meghe Institute of Medical Sciences, Nagpur 442001, India

**Keywords:** oral squamous cell carcinoma, premalignant oral lesions, minichromosome maintenance 3, snuff

## Abstract

Background: Oral squamous cell carcinoma (OSCC) may arise from premalignant oral lesions (PMOL) in most cases. Minichromosome maintenance 3 (MCM3) is a proliferative marker that has been investigated as a potential diagnostic biomarker in the diagnosis of oral cancer. Objectives: To evaluate the association of MCM3 expression, its clinicopathologic parameters and to identify snuff (also called naswar) as a potential risk factor for changes in MCM3 expression in PMOL and OSCC. Methodology: Immunohistochemistry (IHC) of MCM3 was performed on 32 PMOL, 32 OSCC and 16 normal controls after optimization of IHC methodology. Histoscore (0–300) was used as a scoring system and seven different cut-offs were identified for analyses. Data were analyzed using various statistical tests. Results: Among the seven cutoffs, 40% strong positive cells were found to be a better cut-off as they were associated with many pathological variables (Broder’s grade, Aneroth’s grade, and mitotic activity). The differential MCM3 expression in oral lesions (PMOL and OSCC) was statistically significant (*p* = 0.03). Moreover, MCM3 expression is raised with increased duration and frequency of snuff use. Conclusion: High MCM3 expression is associated with disease progression and is a potential indicator of malignant transformations from PMOL to OSCC. Moreover, the use of snuff is associated with MCM3 over-expression.

## 1. Introduction

Cancer of the oral cavity is one of the most frequent cancer types worldwide. More than 95% of head and neck cancers are oral squamous cell carcinomas (OSCC) [1]. It is the third most common malignancy in Pakistan and tenth in the world [2]. It is the eighth most common malignancy affecting men and fifth for women [3]. Males are affected more frequently compared to females (1.5:1) most probably because of high involvement of men in high-risk habits, e.g., smoking, etc., compared to females [4]. According to WHO, oral cancer has a death rate of about 45% for five years after diagnosis. The chances of survival depend on the stage of disease, with poorer prognoses in higher disease stages [5]. 

OSCC has a multifactorial etiology. There is no specific cause, but snuff dipping and alcohol consumption are associated with 90% of patients diagnosed with oral cancer [6]. Snuff is the most common smokeless tobacco used in Pakistan. It is made up of sun and heat dried tobacco leaves, slaked lime, tree bark ash, and flavoring and coloring agents [7].

Most but not all oral cancers precede premalignant oral lesions (PMOL). Among all PMOL the most common are leukoplakia and erythroplakia [8,9] Other potentially malignant conditions that progress to OSCC are lichen planus, submucous fibrosis, and tobacco induced keratosis [10,11].

The diagnosis of PMOL and OSCC is established from clinical and pathological assessment of lesions. Higher grade and end stage disease predicts worse outcomes [12]. However, patients with similar pathological characteristics behave differently, which points to the fact that patients are different at the molecular level. Molecular biomarkers may thus provide a mechanistic insight into tumor biology. Moreover, changes in the genes or proteins involved in carcinogenesis from risk factors, e.g., naswar, may provide further insight into the risk factors associated with the disease.

There are various molecular mechanisms causing excessive proliferation followed by the transformation from normal mucosa to PMOL, and consequently to OSCC [13]. The minichromosome maintenance 3 (MCM3) protein is one of the biological markers used to assess the proliferative activities and regulation of genomic duplications [14]. MCMs are expressed throughout the cell cycle, and their expression is up regulated in proliferating cells, whereas in differentiated and quiescent cells their expression decreases significantly [15].

Since MCM3 identifies both cycling and non-cycling cells with proliferative potential due to its persistence throughout the cell cycle, so antibodies against MCM3 identify more cells in tissue in comparison with other proliferating markers, i.e., Ki-67, the proliferating cell nuclear antigen [16]. Because of its expression in early G1 phase, MCM studies are relevant for determining tumor behavior [17].

Thus, this study aimed to evaluate the potential association of over-expression of MCM3 with tumor pathology. In addition, the expression of MCM3 was evaluated in the differential diagnosis of PMOL and OSCC. Finally, snuff was investigated as a potential risk factor for over-expression of MCM3. The findings of this study are thus anticipated to have both research and clinical implications.

## 2. Materials and Methods

The study was conducted at Institute of Basic Medical Sciences, Khyber Medical University, Khyber College of Dentistry, Peshawar, Rehman Medical Institute, Peshawar, during the year 2018–2019. The sample size was calculated using open Epi software I with assumptions of exposed and unexposed for the outcome of 65% and 30%. Keeping the power at 80%, a sample size of 64 was calculated out of which 32 were PMOL and 32 were OSCC. Sixteen cases of normal oral mucosa were used as normal control. 

Samples were collected according to convenient sampling technique in which formalin fixed paraffin embedded tissue samples from patients diagnosed as having PMOL and OSCC were collected. All patients of any age and gender who came either for diagnostic purposes or surgical treatment were the target population of the study. 

### 2.1. Inclusion Criteria

Patients diagnosed with PMOL and OSCC

Patients who were using or not using naswar

Patients of all age groups and both genders 

### 2.2. Exclusion Criteria

Patients with metastatic tumor in their oral cavities

Patients using smokeless tobacco other than naswar

Patients with neo-adjuvant chemotherapy or chemo-radiation for OSCC in the head and neck region.

### 2.3. Procedure

The study was conducted after approval from the Graduate Study Committee and the Advance Study and Research Board of Khyber Medical University (approval no. DIR/KMU-AS&RB/EM/000579).

A retrospective study of already diagnosed cases of OSCC and PMOL belonging to patients who have provided their consent to be included in research studies, registered in 2011–2017 in the Department of Pathology, Rehman Medical institute and Maxillofacial Surgery Department of Khyber College of Dentistry, were included in the study. The tissues were already processed, and tissue blocks were made according to the standard protocol of tissue processing. The blocks were retrieved according to the PR numbers and lab ID numbers. Eighty-two cases were included in the current study. General information including name, age, and gender were noted from the clinical record of the patient. The sections included 32 cases of OSCC, 32 cases of PMOL, and 16 cases of normal oral mucosa. Two sets of tissue sections of 4 µm were taken from tissue blocks. One set for Hematoxylin and Eosin (H&E) staining and another for immunohistochemistry (IHC) staining were included, in order to investigate the expression of the biomarker. 

### 2.4. Immunostaining

Before performing the final IHC staining, the antibody (MCM3) was optimized to identify preferred IHC parameters as shown in Table 1. The immunostaining was done on a 4µm thick section. After passing through xylene and rehydration in graded alcohol, the tissue sections were immersed in Tris EDTA (pH = 9.0); then, hydrogen peroxide was used to block peroxidase activity. Monoclonal Mouse Anti-Human M7263, clone 101 (DAKO Ltd. (Rochester, NY, USA)), was used followed by secondary antibody. Finally, 3,3′-diaminobenzidine was used for antibody detection. A high grade metastatic squamous cell carcinoma lesion was used as a positive control. PBS was replaced with the primary antibody in order to establish a negative control. The optimal staining achieved with IHC conditions was then used on tissue sections of normal oral mucosa, PMOL and OSCC.

### 2.5. Scoring of Tissue Sections

H&E-stained sections were classified for Broder’s criteria based on histological grades as well-differentiated, moderately differentiated, and poorly differentiated squamous-cell carcinoma. Aneroth’s grading for degree of keratinization, nuclear pleomorphism, and presence of mitotic figures was also observed and recorded [16]. IHC stained tissue sections were scored for both staining intensity and proportion of cells. A histoscore [0 × % positive cells + 1 × positive cells + 2 × positive cells + 3 × positive cells] was generated (range 0–300). Over-expression and under-expression of MCM3 expression was based on a variety of staining cut-offs.

The expression of MCM3 was grouped into over-expression and under-expression according to seven different cut-offs based on the mean value of the recorded histoscore i.e., 92 ± 59 [17], the median histoscore, i.e., 100, 10%, 20%, 30% and 40% strong positive cells [18], and the percentage of the positive tumor cells of any intensity (50%) [17,18,19] (Table 2). 

### 2.6. Statistical Analysis

Data were analyzed using different tests, i.e., Chi-square test/Fisher’s exact test for finding the relation between different variables, i.e., oral lesions, Broder’s grade, Aneroth’s grade, TNM staging, mitotic figures, age, and gender with MCM3 expression. The Spearmen rho test was used for the association between snuff use, its duration (in years), and frequency of snuff use per day with MCM3 expression. Statistical analysis was performed with SPSS software version 21.0 (IBM Corp, Armonk, NY, USA). Statistical significance was set at *p* ≤ 0.05.

## 3. Results

The clinical outcomes of the patients having oral lesions are depicted in Table 3. The mean age at the time of diagnosis was 60 years. Of the total cases, 55 were male and 27 were female with a male: female ratio of 2:1. Site distribution in the selected patients showed cheek mucosa as the predominant site followed by alveolar mucosa.

**Figure 1:**
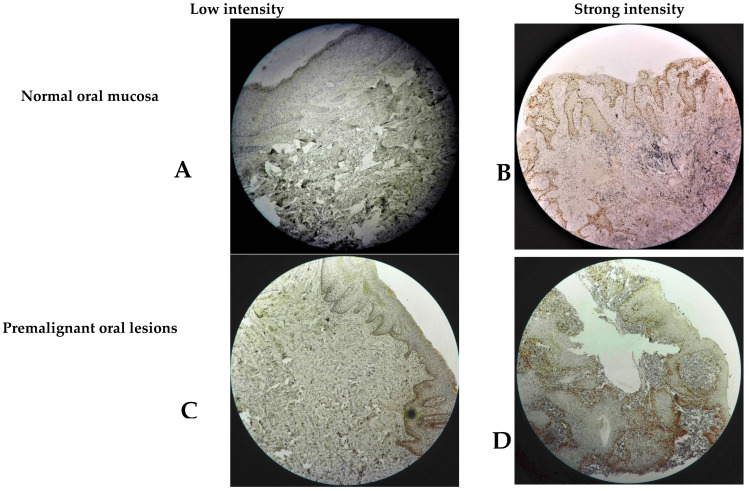

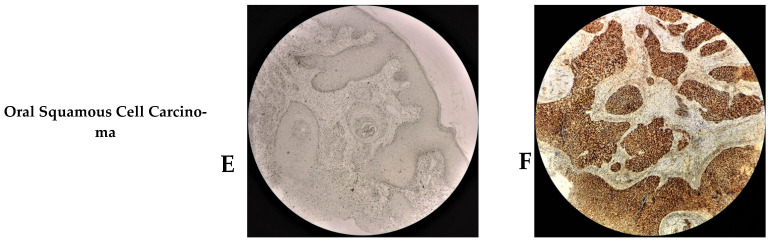
Staining intensity of proliferative oral epithelial cells showing weak and strong staining of proliferating cells of different oral lesions at the mean 92 cut-off. (**A**,**B**): low and strong intensity MCM3 expression in normal oral mucosa, respectively. (**C**,**D**): low and strong intensity MCM3 expression in PMOL, respectively. (**E**,**F**): low and strong intensity MCM3 expression in OSCC, respectively.

MCM3 expression was evaluated based on the intensity and proportion of staining (Figure 1). The relationship between different clinical and histopathological parameters and MCM3 expression was evaluated based on seven IHC cut-offs, i.e., mean, median, 10% strong positive cells, 20% strong positive cells, 30% strong positive cells, 40% strong positive cells, and the percentage of positive tumor cells. The cut-off, 40% strong positive cells, was found to have a better relationship with clinicopathological variables. Based on IHC scoring, 40% and above strong positive tumor cells would be regarded as over-expression and below 40% as under-expression.

The results showed that at different cut-offs, all the normal cases were under-expressed for nucleic expression of MCM3, while in the case of PMOL (n = 34), including leukoplakia, erythroplakia, lichen planus, submucous fibrosis, and tobacco-induced keratosis, under-expression is observed in most of the cases. For the OSCC, at all cut-offs, there was an evident over-expression of nuclear positivity for MCM3, which is spread from the basal to the superficial layer of the epithelium, except at 30% and 40% cut-offs that showed under-expression. A statistically significant difference was observed in the expression level of MCM3 among normal, PMOL, and OSCC (*p* = 0.03). There were 32 cases of OSCC. According to Broder’s criteria, 24 cases were graded as well differentiated SCC, 7 cases were moderately differentiated, and only 1 case was graded as poorly differentiated SCC. When the Chi-square test was applied at different set cut-offs there was an over-expression of the MCM3 in most of the cases of well, moderate and poorly differentiated SCC. Considering the 40% strong positive cells, there was a statistically significant difference in the under-expression and over-expression categories of MCM3 (*p* = 0.02) regarding Broder’s grade. The results showed that out of the total 32 cases of OSCC, 16 cases were graded as grade I, 15 cases were grade II and only 1 case was graded as grade III as per the Aneroth’s classification. When the Chi-square test was applied at different set cut-offs, there was an over-expression of the MCM3 in most of the cases of Aneroth grades I, II, and III of SCC, and very few cases were under-expressed for MCM3. The results depicted a significant correlation between MCM3 expression and Aneroth’s grade at 40% strong positive cell cut-off (*p* = 0.023) (Table 4, Table 5, Table 6 and Table 7).

Based on TNM staging criteria, it was observed that 17 cases of OSCC were staged as T1 stage, 4 cases as T2, 9 cases as T3 and only 2 cases were staged as T4. When Chi-square test was applied at different set cut-offs, there was an over-expression of the MCM3 in most of the cases of TNM stages T1, T2, T3 and T4 of SCC, and very few cases were under expressed for MCM3. The MCM3 expression was not statistically significant in association with TNM stage (*p* = 0.83) (Table 8).

Of the total cases of OSCC, 27 cases were scored as having 0-3 mitotic figures, while 5 cases were scored with ≥4 mitotic figures. When the Chi-square test was applied at different set cut-offs, there was an over-expression of the MCM3 in most of the cases. These results showed that there was a significant correlation between MCM3 expressions, except at 40%, because mitotic figures were statistically associated with expression levels of MCM3 (*p* = 0.008) (Table 9).

MCM3 expression was tested with snuff (naswar) users and non-snuff users based on the frequency of snuff use and its duration (in years). The statistical results showed that a weak to moderate association was found between snuff use, its duration and frequency with MCM3 expression (*p* = 0.015, 0.004 and 0.027, respectively, at 40% strong positive cells cut-off) (Table 10, Table 11 and Table 12).

## 4. Discussion

Many studies correlating histopathological and clinical parameters with OSCC have been done; still further studies, including more comprehensive materials and methods, are needed to clarify the importance of these parameters. Moreover, it is generally accepted that non-invasive precursor lesions can lead to oral cancer development, however it is still uncertain how OSCC develops from premalignant lesions [19]. The present study focuses on the differential expression of MCM3 in normal oral mucosa, PMOL and OSCC tissue samples. The relation between MCM3 expression was assessed with various clinico-pathological parameters (TNM stage, Broder’s grade, Aneroth grade, mitotic figures, etc.) and snuff use (duration and frequency). 

In the present study, MCM3 expression showed a tendency to be stronger for control through PMOL and significantly upregulated in OSCC, showing from no staining to very weak staining in normal oral mucosa samples, weak to moderate staining in PMOL, and moderate to strong staining in OSCC samples. These results agree with Rezvani G et al. (2015) [20] and Gan N et al. (2010) [21], who also showed differential expression of MCM3 in control, premalignant, and in OSCC cases. These results indicate that MCM3 over-expression carries significant diagnostic potential in premalignant lesions, including leukoplakia, erythroplakia, lichen planus, submucous fibrosis, and tobacco associated keratosis that have a potential risk for transformation into OSCC.

In order to improve the precision of morphological criteria, we have used Broder’s and Aneroth’s grading system. Our observed results showed a significant association of MCM3 over-expression with increasing Broder’s histological grade using two out of seven cut-offs, including 40% strong positive cells and percentage of positive cells. Increasing Aneroth’s histological grade in OSCCs was also found to be significantly associated with MCM3 over-expression using 40% strong positive cells as a cut-off. The gradual increase in MCM3 expression with disease grade agrees with Gan N et al. (2010), ref. [21] suggesting that there is a gradual loss of MCM3 expression in cells undergoing differentiation. Thus, without the differentiated cells, the proliferative activity and MCM3 expression will increase. This can be taken as a diagnostic factor for predicting the severity of the disease. Future evidence-based in vitro research may be done on cell lines of oral cancer and human oral cancer cell lines to further observe this type of association of MCM3 in OSCC cases.

Regarding TNM staging and MCM3 expression, literature shows an association between tumor size and lymph node metastasis as noted by Yu G et al. (2008) [22]. The difference between our study and the positive studies may be due to the difference in behaviour of MCM family proteins. Our results agreed with Lopes VKM et al. (2017) [23], showing no significant relation between TNM staging and MCM3 expression assessed. Therefore, the role of MCM3 expression in evaluating disease severity remains unclear.

The proliferation is one of the basic hallmarks of cancer. Mitotic count is considered a reproducible means of proliferative activity assessment. Very little work is present on this parameter regarding oral cancer. MCM3 overexpression was found to be associated with an increased number of mitotic figures using two cut-offs, which include 40% strong positive cells and a percentage of positive cells. This suggests that MCM3 overexpression might be an indicator of proliferating activity in malignant squamous epithelial cells. Since the marker used in our study is a proliferative biomarker, mitotic figure relation is of utmost importance.

The correlation between clinical parameters, i.e., age and gender, was tested, showing insignificant results. These results agreed with Lopes VKM et al. (2017) [23].

Since snuff is considered a risk factor for the progression of cancer, it was investigated along with MCM3 expression to observe any association between the two variables. Our result showed significant association at 20%, 30%, and 40% strong positive cells, showing that there is a moderate positive association between naswar and MCM3 expression. This indicates that there is an increased MCM3 expression in snuff users. The association between the duration of snuff use and MCM3 expression showed moderate positivity at 10%, 20%, 30%, and 40%, showing that increased duration of snuff use also increases MCM3 expression. The result of frequency of snuff use and MCM3 shows significant association at 40% strong positive cells. This moderate-strong positive association between MCM3 over-expression in OSCCs and frequency of snuff used per day indicates that snuff use is related to increased proliferative activity via MCM3 over-expression. Therefore, snuff use might be considered a potential risk factor for changes in MCM3 expression in PMOL and OSCC. There has been no work done in literature regarding the association between these two parameters; therefore, further work is needed.

## 5. Limitations

This study had certain constraints that might have an impact on the interpretation of our research findings to some extent.

i. The main study design limitation is the total number of OSCC cases. It affects the data analysis and the results implementation in the population.
ii. Regarding data, there were very little premalignant and cancer registries in our hospitals. The clinical data, especially of naswar use and its type from patients, is incomplete.
iii. Survival analysis was not possible due to the limited availability of survival data.
iv. Regarding future research, additional in vitro studies on human oral cancer cell lines would be needed to further confirm the association of MCM3 with oral cancer.

## 6. Conclusions

In conclusion, the stepwise increasing differential expression of MCM3 from normal oral mucosa to PMOL and OSCC samples in our study points towards the potential role of MCM3 in malignant transformation. Moreover, the association between snuff use and MCM3 over-expression in our OSCC samples reveals the potential molecular role of snuff in the over-expression of MCM3 protein and carcinogenesis. The increased expression level of MCM3 and its association with high mitotic activity further shows that the assessment of this protein could prove useful as a diagnostic marker in OSCCs. 

Thus, we conclude from our study that MCM3 could be a valuable marker in differential diagnosis of premalignant oral lesions and oral squamous cell carcinoma. It could be considered a surrogate marker of malignant transformation. The study indicates the probability of the application of MCM3 for molecular targeted therapy in diseased patients.

## Figures and Tables

**Table 1:** Final immunohistochemistry parameters set on archival tissue sections for MCM.

Antibody	Company	Clone	Antigen Retrieval	pH	Antibody Dilution	Retrieval Time	Incubation Temp. (°C)	Incubation Time (h)	Incubation Method
MCM3	DAKO	101	Heat induced epitope retrieval (HIER)	Tris EDTA pH = 9.0	1/50	1 h	100	1 h	Oven

**Table 2:** Different set cut-offs for data analysis.

Statistical Method	Cut-Off	
	Over-Expression	Under-Expression
Mean	>92	≤92
Median	>100	≤100
10% strong positive cells	>10%	≤10%
20% strong positive cells	>20%	≤20%
30% strong positive cells	>30%	≤30%
40% strong positive cells	>40%	≤40%
Percentage of positive cells	>50%	≤50%

**Table 3:** Demographic parameters and their observations.

Demographic		Observation (N = 82)
Age	≤60 >60	58 34
Gender	Male Female	55 27
Location of tumors	Cheek mucosa/sulcus Gingival/alveolar mucosa Tongue Lip Floor of the mouth Palate	28 18 14 7 4 2
Oral lesions	Normal PMOL OSCC	16 34 32

**Table 4:** Differential MCM3 expression and oral lesions.

Statistical Method	MCM3 Over-Expression	MCM3 Under-Expression	*p*-Value
Mean	Normal: 0 PMOL: 14 OSCC: 29	Normal: 16 PMOL: 20 OSCC: 03	0.000 *
Median	Normal: 0 PMOL: 12 OSCC: 30	Normal: 16 PMOL: 22 OSCC: 02	0.000 *
10% strong positive cells	Normal: 0 PMOL: 10 OSCC: 28	Normal: 16 PMOL: 24 OSCC: 04	0.000 *
20% strong positive cells	Normal: 0 PMOL: 0 OSCC: 25	Normal: 16 PMOL: 34 OSCC: 07	0.000 *
30% strong positive cells	Normal: 0 PMOL: 0 OSCC: 14	Normal: 16 PMOL: 34 OSCC: 18	0.000 *
40% strong positive cells	Normal: 0 PMOL: 0 OSCC: 04	Normal: 16 PMOL: 34 OSCC: 28	0.037 *
Percentage of positive cells	Normal: 0 PMOL: 1 OSCC: 17	Normal: 16 PMOL: 33 OSCC: 15	0.000 *

* Statistically significant.

**Table 5:** MCM3 expression correlation with clinicopathologic parameters at 40% strong positive cells cut-off.

Parameters	MCM3 Over-Expression	MCM3 Under-Expression	*p*-Value
Oral lesions Normal PMOL OSCC	0 0 04	16 34 28	0.03 *
Broder’s grade Well diff: OSCC Moderately diff: OSCC Poorly diff: OSCC	2 1 1	22 6 0	0.02 *
Aneroth’s grade Grade I Grade II Grade III	1 2 1	15 13 0	0.02 *
TNM stage Stage I Stage II Stage III Stage IV	2 1 1 0	15 3 8 2	0.83
Mitotic figures (0–3) (≥4)	1 3	26 2	0.008 *

* Statistically significant.

**Table 6:** MCM3 correlation with Broder’s grading system.

Statistical Method	MCM3 Over-Expression	MCM3 Under-Expression	*p*-Value
Mean	Well diff SCC: 23 Moderately diff SCC: 6 Poorly diff SCC: 1	Well diff SCC: 1 Moderately diff SCC: 1 Poorly diff SCC: 0	0.602
Median	Well diff SCC: 23 Moderately diff SCC: 6 Poorly diff SCC: 1	Well diff SCC: 1 Moderately diff SCC: 1 Poorly diff SCC: 0	0.602
10% strong positive cells	Well diff SCC: 22 Moderately diff SCC: 5 Poorly diff SCC: 1	Well diff SCC: 2 Moderately diff SCC: 2 Poorly diff SCC: 0	0.337
20% strong positive cells	Well diff SCC: 19 Moderately diff SCC: 5 Poorly diff SCC: 1	Well diff SCC: 5 Moderately diff SCC: 2 Poorly diff SCC: 0	0.787
30% strong positive cells	Well diff SCC: 10 Moderately diff SCC: 3 Poorly diff SCC: 1	Well diff SCC: 14 Moderately diff SCC: 4 Poorly diff SCC: 0	0.514
40% strong positive cells	Well diff SCC: 2 Moderately diff SCC: 1 Poorly diff SCC: 1	Well diff SCC: 22 Moderately diff SCC: 6 Poorly diff SCC: 0	0.025 *
Percentage of positive cells	Well diff SCC: 10 Moderately diff SCC: 6 Poorly diff SCC: 1	Well diff SCC: 14 Moderately diff SCC: 1 Poorly diff SCC: 0	0.077

* Statistically significant.

**Table 7:** MCM3 expression correlation with Aneroth’s grading system.

Statistical Method	MCM3 Over-Expression	MCM3 Under-Expression	*p*-Value
Mean	Grade I: 16 Grade II: 13 Grade III: 1	Grade I: 0 Grade II: 2 Grade III: 0	0.299
Median	Grade I: 16 Grade II: 13 Grade III: 1	Grade I: 0 Grade II: 2 Grade III: 0	0.299
10% strong positive cells	Grade I: 15 Grade II: 12 Grade III: 1	Grade I: 1 Grade II: 3 Grade III: 0	0.476
20% strong positive cells	Grade I: 1 Grade II: 14 Grade III: 10	Grade I: 2 Grade II: 5 Grade III: 0	0.324
30% strong positive cells	Grade I: 8 Grade II: 5 Grade III: 1	Grade I: 8 Grade II: 10 Grade III: 0	0.333
40% strong positive cells	Grade I: 1 Grade II: 2 Grade III: 1	Grade I: 15 Grade II: 13 Grade III: 0	0.023 *
Percentage of positive cells	Grade I: 7 Grade II: 9 Grade III: 1	Grade I: 9 Grade II: 6 Grade III: 0	0.421

* Statistically significant.

**Table 8:** MCM3 expression correlation with TNM stage.

Statistical Method	MCM3 Over-Expression	MCM3 Under-Expression	*p*-Value
Mean	Stage I: 17 Stage II: 4 Stage III: 7 Stage IV: 2	Stage I: 0 Stage II: 0 Stage III: 2 Stage IV: 0	0.142
Median	Stage I: 17 Stage II: 4 Stage III: 7 Stage IV: 2	Stage I: 0 Stage II: 0 Stage III: 2 Stage IV: 0	0.142
10% strong positive cells	Stage I: 16 Stage II: 4 Stage III: 6 Stage III: 2	Stage I: 1 Stage II: 0 Stage III: 3 Stage IV: 0	0.164
20% strong positive cells	sStage I: 15 Stage II: 4 Stage III: 6 Stage IV: 0	Stage I: 2 Stage II: 0 Stage III: 3 Stage IV: 2	0.019 *
30% strong positive cells	Stage I: 7 Stage II: 3 Stage III: 4 Stage IV: 0	Stage I: 10 Stage II: 1 Stage III: 5 Stage IV: 2	0.363
40% strong positive cells	Stage I: 2 Stage II: 1 Stage III: 1 Stage IV: 0	Stage I: 15 Stage II: 3 Stage III: 8 Stage IV: 2	0.830
Percentage of positive cells	Stage I: 9 Stage II: 3 Stage III: 3 Stage IV: 2	Stage I: 8 Stage II: 1 Stage III: 6 Stage IV: 0	0.267

* Statistically significant.

**Table 9:** MCM3 expression correlation with mitotic figures.

Statistical Method	MCM3 Over-Expression	MCM3 Under-Expression	*p*-Value
Mean	Mitotic figure (0–3): 25 Mitotic figure (≥4): 5	Mitotic figure (0–3): 2 Mitotic figure (≥4): 0	1.000
Median	Mitotic figure (0–3): 25 Mitotic figure (≥4): 5	Mitotic figure (0–3): 2 Mitotic figure (≥4): 0	1.000
10% strong positive cells	Mitotic figure (0–3): 24 Mitotic figure (≥4): 4	Mitotic figure (0–3): 3 Mitotic figure (≥4): 1	0.512
20% strong positive cells	Mitotic figure (0–3): 21 Mitotic figure (≥4): 4	Mitotic figure (0–3): 6 Mitotic figure (≥4): 1	1.000
30% strong positive cells	Mitotic figure (0–3): 11 Mitotic figure (≥4): 3	Mitotic figure (0–3): 16 Mitotic figure (≥4): 2	0.631
40% strong positive cells	Mitotic figure (0–3): 1 Mitotic figure (≥4): 3	Mitotic figure (0–3): 26 Mitotic figure (≥4): 2	0.008 *
Percentage of positive cells	Mitotic figure (0–3): 12 Mitotic figure (≥4): 5	Mitotic figure (0–3): 15 Mitotic figure (≥4): 0	0.046 *

* Statistically significant.

**Table 10:** Snuff use association with MCM3 expression.

Statistical Method	Correlation Coefficient		*p*-Value
	Snuff Use (1 = Yes, 2 = No)	MCM3 Expression	
Mean	1	0.192	0.152
	0.192	1	
Median	1.000	0.159	0.238
	0.159	1.000	
Percentage of positive cell	1.000	0.225	0.09
	0.225	1.000	
10% strong positive cells	1.000	0.151	0.263
	0.151	1.000	
20% strong positive cells	1.000	0.330	0.012 *
	0.330	1.000	
30% strong positive cells	1.000	0.364	0.005 *
	0.364	1.000	
40% strong positive cells	1.000	0.322	0.015 *
	0.322	1.000	

* Statistically significant.

**Table 11:** Association of duration of snuff use with MCM3 expression.

Statistical Method	Correlation Coefficient		*p*-Value
	Duration of Snuff Use (Years)	MCM3 Expression	
Mean	1.000	0.301	0.163
	0.301	1.000	
Median	1.000	0.301	0.163
	0.301	1.000	
Percentage of positive cells	1.000	−0.158	0.460
	−0.158	1.000	
10% strong positive cells	1.000	0.360	0.084
	0.360	1.000	
20% strong positive cells	1.000	0.358	0.094
	0.358	1.000	
30% strong positive cells	1.000	0.457	0.028 *
	0.457	1.000	
40% strong positive cells	1.000	0.581	0.004 *
	0.581	1.000	

* Statistically significant.

**Table 12:** Association of frequency of snuff use with MCM3 expression.

Statistical Method	Correlation Coefficient		*p*-Value
	Frequency of Snuff Use/Day	MCM3 Expression	
Mean	1.000	0.277	0.211
	0.277	1.000	
Median	1.000	0.277	0.211
	0.277	1.000	
Percentage of positive cell	1.000	−0.223	0.307
	−0.223	1.000	
10% strong positive cells	1.000	0.227	0.211
	0.227	1.000	
20% strong positive cells	1.000	0.204	0.362
	0.204	1.000	
30% strong positive cells	1.000	0.325	0.139
	0.325	1.000	
40% strong positive cells	1.000	0.471	0.027 *
	0.471	1.000	

* Statistically significant.

## Data Availability

The data set used in the current study will be made available on request from Rabia Zahir; rabiahaseeb44@gmail.com.

## References

[1] Kumar V., Abbas A.K., Fausto N., Aster J.C. (2014). Robbins and Cotran Pathologic Basis of Disease, Professional Edition e-Book.

[2] Lippman S.M., Hong W.K. (2001). Molecular markers of the risk of oral cancer. N. Engl. J. Med..

[3] Feller L., Lemmer J. (2012). Oral squamous cell carcinoma: Epidemiology, clinical presentation and treatment. J. Cancer Ther..

[4] Panzarella V., Pizzo G., Calvino F., Compilato D., Colella G., Campisi G. (2014). Diagnostic delay in oral squamous cell carcinoma: The role of cognitive and psychological variables. Int. J. Oral. Sci..

[5] Johnson N. (2001). Tobacco use and oral cancer: A global perspective. J. Dent. Educ..

[6] Imam S.Z., Nawaz H., Sepah Y.J., Pabaney A.H., Ilyas M., Ghaffar S. (2007). Use of smokeless tobacco among groups of Pakistani medical students—A cross sectional study. BMC Public Health.

[7] Zini A., Czerninski R., Sgan-Cohen H.D. (2010). Oral cancer over four decades: Epidemiology, trends, histology, and survival by anatomical sites. J. Oral. Pathol. Med..

[8] Rivera C., Venegas B. (2014). Histological and molecular aspects of oral squamous cell carcinoma (Review). Oncol. Lett..

[9] Warnakulasuriya S., Johnson N.W., van der Waal I. (2007). Nomenclature and classification of potentially malignant disorders of the oral mucosa. J. Oral. Pathol. Med..

[10] Van der Waal I. (2009). Potentially malignant disorders of the oral and oropharyngeal mucosa; terminology, classification and present concepts of management. Oral. Oncol..

[11] Pindborg J.J., Reichart P.A., Smith C.J., Van der Waal I. (1997). Definitions and Explanatory Notes. Histological Typing of Cancer and Precancer of the Oral Mucosa.

[12] Hsue S.S., Wang W.C., Chen C.H., Lin C.C., Chen Y.K., Lin L.M. (2007). Malignant transformation in 1458 patients with potentially malignant oral mucosal disorders: A follow-up study based in a Taiwanese hospital. J. Oral. Pathol. Med..

[13] Musahl C., Holthoff H.P., Lesch R., Knippers R. (1998). Stability of the replicative Mcm3 protein in proliferating and differentiating human cells. Exp. Cell Res..

[14] Tachibana K.E., Gonzalez M.A., Coleman N. (2005). Cell-cycle-dependent regulation of DNA replication and its relevance to cancer pathology. J. Pathol..

[15] Ur Rahaman S.M., Ahmed Mujib B. (2014). Histopathological correlation of oral squamous cell carcinoma among younger and older patients. J. Oral. Maxillofac. Pathol..

[16] Lee Y.S., Ha S.A., Kim H.J., Shin S.M., Kim H.K., Kim S., Kang C.S., Lee K.Y., Hong O.K., Lee S.H. (2010). Minichromosome maintenance protein 3 is a candidate proliferation marker in papillary thyroid carcinoma. Exp. Mol. Pathol..

[17] Carreón-Burciaga R.G., González-González R., Molina-Frechero N., Bologna-Molina R. (2015). Immunoexpression of Ki-67, MCM2, and MCM3 in ameloblastoma and ameloblastic carcinoma and their correlations with clinical and histopathological patterns. Dis. Markers.

[18] Ali A., Brown V., Denley S., Jamieson N.B., Morton J.P., Nixon C., Graham J.S., Sansom O.J., Carter C.R., McKay C.J. (2014). Expression of KOC, S100P, mesothelin and MUC1 in pancreatico-biliary adenocarcinomas: Development and utility of a potential diagnostic immunohistochemistry panel. BMC Clin. Pathol..

[19] Mithani S.K., Mydlarz W.K., Grumbine F.L., Smith I.M., Califano J.A. (2007). Molecular genetics of premalignant oral lesions. Oral Dis..

[20] Rezvani G., Andisheh-Tadbir A., Ashraf M.J., Amanpour S., Kamali F., Fardisi S. (2015). Evaluation of Minichromosome Maintenance-3 (MCM3) in Oral Squamous Cell Carcinoma. J. Dent..

[21] Gan N., Du Y., Zhang W., Zhou J. (2010). Increase of Mcm3 and Mcm4 expression in cervical squamous cell carcinomas. Eur. J. Gynaecol. Oncol..

[22] Yu G., Wang J., Chen Y., Wang X., Pan J., Li Q., Xie K. (2008). Tissue microarray analysis reveals strong clinical evidence for a close association between loss of annexin A1 expression and nodal metastasis in gastric cancer. Clin. Exp. Metastasis.

[23] Lopes V.K., Jesus A.S., Souza L.L., Miyahara L.A., Guimarães D.M., Pontes H.A., Pontes F.S., Carvalho P.L. (2017). Ki-67 protein predicts survival in oral squamous carcinoma cells: An immunohistochemical study. Braz. Oral. Res..

